# Semi-Automated Therapeutic Drug Monitoring as a Pillar toward Personalized Medicine for Tuberculosis Management

**DOI:** 10.3390/pharmaceutics14050990

**Published:** 2022-05-05

**Authors:** Rannissa Puspita Jayanti, Nguyen Phuoc Long, Nguyen Ky Phat, Yong-Soon Cho, Jae-Gook Shin

**Affiliations:** 1 Center for Personalized Precision Medicine of Tuberculosis, Inje University College of Medicine, Busan 47392, Korea; rannissapuspita15@gmail.com (R.P.J.); phuoclong@inje.ac.kr (N.P.L.); ngkyphat@live.inje.ac.kr (N.K.P.); ysncho@gmail.com (Y.-S.C.); 2 Department of Pharmacology and Pharmacogenomics Research Center, Inje University College of Medicine, Busan 47392, Korea; 3 Department of Clinical Pharmacology, Inje University Busan Paik Hospital, Busan 47392, Korea

**Keywords:** personalized medicine, tuberculosis, therapeutic drug monitoring, model-informed precision dosing, clinical decision support system

## Abstract

Standard tuberculosis (TB) management has failed to control the growing number of drug-resistant TB cases worldwide. Therefore, innovative approaches are required to eradicate TB. Model-informed precision dosing and therapeutic drug monitoring (TDM) have become promising tools for adjusting anti-TB drug doses corresponding with individual pharmacokinetic profiles. These are crucial to improving the treatment outcome of the patients, particularly for those with complex comorbidity and a high risk of treatment failure. Despite the actual benefits of TDM at the bedside, conventional TDM encounters several hurdles related to laborious, time-consuming, and costly processes. Herein, we review the current practice of TDM and discuss the main obstacles that impede it from successful clinical implementation. Moreover, we propose a semi-automated TDM approach to further enhance precision medicine for TB management.

## 1. Introduction

Despite numerous efforts to end tuberculosis (TB) over the past several decades, TB remains a major issue in global health and infectious disease-related mortality [1]. The World Health Organization (WHO) estimated almost 10 million new cases of TB in 2020 [1]. Standard treatment has failed to control the disease efficiently. Its significance was highlighted by the enormous number of deaths in that year, approximately 1.5 million, which was reported as the first increase in more than a decade [1]. Moreover, the 2019 coronavirus (COVID-19) pandemic has interfered with patients’ ability to obtain sufficient treatment [2]. The pandemic may potentially contribute to the growing incidence of multi-drug resistant (MDR) TB. Therefore, the elimination of TB requires more innovative approaches.

Precision medicine is an evolving approach to treating and preventing disease by taking into account individual variability in genes, the clinical situation, demographics, environment, and lifestyle [3,4]. The pillar of this approach is that different drug responses to treatment are due to inter-individual variability (IIV) [4,5]. IIV in drug response is an issue in clinical practice since it is difficult to predict who will be treated successfully and who will suffer from an adverse drug reaction (ADR) and treatment failure [4,6]. Patients who belong to edge populations, such as those with renal or hepatic impairments, critically ill patients, and patients taking concomitant drugs, will benefit more from precision medicine [7]. Therefore, precision medicine is considered a promising approach regarding optimizing current anti-TB drug dose regimens to ensure safe and effective treatment [8,9].

Model-informed precision dosing (MIPD) has become a promising tool in implementing precision medicine, particularly in TB treatment [4,10,11]. This tool can support the adjustment of anti-TB drug doses to correspond with individual pharmacokinetic (PK) profiles and to assist in therapeutic drug monitoring (TDM) implementation [8,12,13]. In addition, TDM may help generate essential data on the pharmacokinetics–pharmacodynamics (PK/PD) of the treatment to better understand the underlying reasons for IIV in drug exposures [10,14]. TDM is usually implemented only when the treatment fails or an ADR appears in clinical practice [14,15]. Due to the rapid progression of the TB disease, it is necessary to ensure effective treatment. Thus, TDM should be an indispensable part of the treatment plan of TB patients and should be utilized in routine practice to optimize drug exposure from the beginning of treatment [14,16]. Recently, many lines of evidence have suggested a potentially important role of TDM for TB treatment in the era of precision medicine [8,12,17,18]. For instance, a previous case-series study showed that patients with delayed sputum conversion and complicated clinical situations had improved treatment response following dose adjustment according to the TDM result [18]. The measured concentration showed a low value compared to the target therapeutic range of at least Rifampicin (RIF), the key drug of TB treatment [18]. This study has shown the clinical advantage of TDM at the bedside, which can be helpful for TB eradication.

Conventional TDM is applied by taking the blood at the peak concentration and comparing this measured concentration with the therapeutic reference range [12,17]. Nonetheless, this approach has a disadvantage. The clinical outcome of anti-TB drugs is associated with drug exposure, rather than maximum concentration achieved [14,19]. Hence, frequent sampling is required to calculate this observed exposure in order to obtain a reliable measure of exposure to anti-TB drugs in conventional TDM practice [14,20]. This method requires a lot of time and human resources [20]. Limited sampling strategies (LSS) have been proposed to predict exposure with high precision while using fewer sampling points [21,22]. This strategy will improve the convenience of PK sampling for patients. A two-sampling-time scheme (2- and 6-h post-dose) for most anti-TB drugs is generally considered [12,17]. However, this sampling scheme is not applicable for some anti-TB drugs, such as rifabutin, which is better represented by a 3 h and 7 h post-dose scheme, and rifapentine and para-aminosalicylic (PAS), which have better peak concentrations at 5 h and 6 h samples, respectively [12,17]. The integration of the observed drug concentration with model-informed precision dosing in order to provide individual dosage using Bayesian forecasting has made this approach more accessible [23,24]. Despite all the conveniences that were recently established, the application of TDM remains limited to some centralized and high-standard facilities [16,25]. Several hurdles related to costly, laborious, and time-consuming processes have restricted access to TDM for most TB patients, particularly those in developing countries [14,16]. Recent developments in advanced technological and computational approaches have shown potential in improving TDM to bring it closer to the patient [4,10,25]. In this article, we review the current practice of TDM and discuss the main obstacles that hamper it from successful clinical implementation. Moreover, we propose a novel strategy to further enhance precision medicine for TB management through the innovation of the TDM process.

## 2. Overview of Therapeutic Drug Monitoring in TB Treatment

Key aspects of successful TB treatment have been identified as patients’ adherence and adequate drug exposure, particularly to the key drugs of treatment (INH, RIF, PZA) [26,27]. In addition to direct observed therapy, short-course strategy (DOTs), the latest ATS guidelines recommend TDM in patients infected with *Mycobacterium tuberculosis* with the possibility of poor prognosis or at risk for altered drug exposure [27]. Nevertheless, TDM implementation is seldom used in clinical settings, and guidelines do not specify PK/PD target ranges. The potential for underdosing with the standard regimen has raised concerns regarding the effectiveness of the current regimen. Many studies have reported that the current doses for first-line anti-TB drugs used internationally provide low exposure to many patients [28]. On the other hand, high concentrations of anti-TB drugs (such as linezolid (LZD), PZA, INH, cycloserine (CS), bedaquiline (BDQ), clofazimine (CFZ), ethambutol (EMB), and moxifloxacin (MFX)) were suggested to contribute to the increased risk of ADR [17,29,30]. Treatment interruptions, drug replacements, prolonged treatment length, hospitalization, low compliance, and increased resistance to TB drugs may be caused by adverse events during TB treatment [31]. The difference in exposure of the current standard dose among TB patients is prominently caused by IIV [32]. We also present evidence regarding the clinically relevant concerns regarding altered drug exposure in Table 1.

To overcome the problems mentioned above, TDM has been advised as a solution in order to maintain the therapeutic concentrations of anti-TB drugs within a desired range. In this section, we will discuss the currently available approaches to interpreting TDM data.

### 2.1. Conventional TDM

In conventional TDM, the drug concentration measured is interpreted by referring to the therapeutic range [20]. The therapeutic range is defined as the concentration range that can treat the disease safely and effectively [17,45]. Conventional TDM has several advantages, including direct implementation and uncomplicated interpretation [20]. Despite these advantages, conventional TDM is a laborious process [16]. By monitoring the drug concentration at a steady state, the contribution of PK variability to differences in dose requirements can be evaluated [46]. Thus, the sample should be taken in a steady state. Another weakness of conventional TDM is the use of one representative measured concentration as a surrogate for the overall drug exposure [47]. In anti-TB drugs, peak concentration (C_max_) is frequently used to interpret therapeutic range, or trough concentration (C_min_) is associated with the exposure [45,48]. Sample timing is critical in conventional TDM [20,49], and the sample should be taken within the time window in order to be interpreted accurately [17,25,50]. Lastly, using therapeutic range as the target of treatment monitoring may lead to suboptimal attainment of the PK/PD target. This is especially true for drugs with a wide therapeutic range, such as RIF and PZA [12,15,17]. Moreover, the dose adjustment performed in this approach has no clear guidelines [25,50,51]. The dose adjustment is usually conducted according to the physician’s judgment [18,51,52] or is generally made based on a mathematical “rule of three”, changing either the dose or dosing interval (Dettli’s rule) [53]. The rule of three, known as Dettli’s rule, is the rule for calculation of dose adjustment or dosing interval adjustment based on creatinine clearance. It is mostly used in patients with renal impairment [20]. In Dettli’s rule, drug half-lives are not taken into account and the possibility of nonrenal elimination changes is not considered [53]. For drugs that are primarily eliminated through renal elimination, Dettli’s rule allows for reasonable individualization of drug dosing [54]. Nevertheless, the right rule cannot be chosen based on pharmacokinetic principles alone, but rather also on pharmacodynamic principles [54].

### 2.2. Model-Informed Precision-Dosing-Based TDM

**Figure 1:**
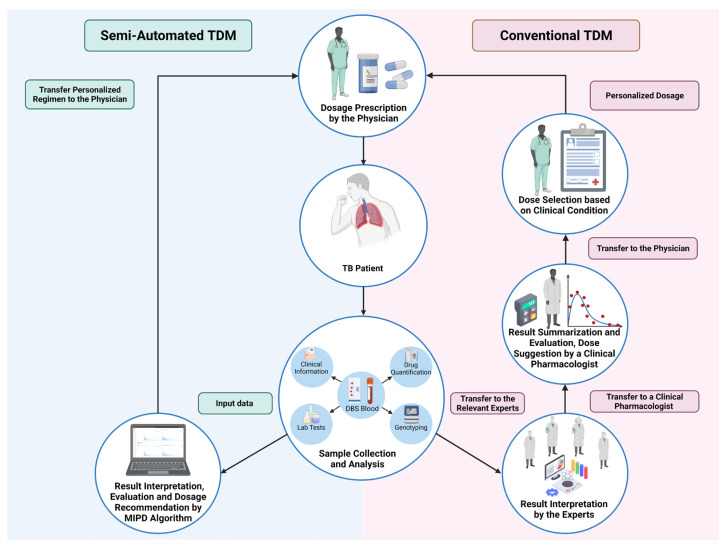
Comparison of (left) the semi-automated TDM workflow and (right) the conventional TDM workflow. The conventional TDM workflow also presents all steps in the TDM process. TDM: therapeutic drug monitoring; TB: tuberculosis.

Model-informed precision dosing (MIPD) is a concept that covers the application of mathematical models and simulation to predict individual tailored doses [55,56]. Applying MIPD in TDM aims to improve drug therapy and achieve better patient care by considering the patient’s characteristics and IIV [20,56]. MIPD in TDM was associated with implementing population PK models [4,10]. A population PK model has several components: a structural model describing the typical PK value, covariates defining the relationship between individual PK parameters and specific covariates, the mathematical value of IIV, and residual variability of the model [57]. It is worth mentioning that the population PK model can comprehensively evaluate the magnitude of the covariates’ influence on the PK parameter, consider multiple covariates during the analysis, and estimate PK parameters such as the area under concentration (AUC) [14,58]. The model can predict the dosing regimen that enhances the possibility of achieving the PK/PD or toxicity targets using the individual PK parameters [59,60,61]. Bayesian forecasting is widely recognized as the best conceptual framework for implementing TDM and is available via various computer tools [56,62]. Bayesian forecasting can be used to derive the individual PK parameters from the measured concentration and estimated the appropriate drug exposure for each individual, even if only using one single point [20,59,63]. The samples can be taken anytime, and a steady state may not be required [20]. MIPD and the individual PK parameter estimates’ precision typically increase as more drug concentration samples are added [64,65]. Lastly, via the gradual improvement of medical technology systems, it is possible that in the near future MIPD will be directly implemented into electronic medical record systems [66]. Thus, the possible errors and time-consuming processes will be removed during data input into the MIPD software. Semi-automated TDM is a thorough approach that includes MIPD as a key step. Although the approach still requires some earlier steps to be conducted by human resources, MIPD will be integrated into an algorithm for generating automated result interpretation, dosing recommendations, and TDM reports for each patient. The outline of the comparison between conventional TDM and semi-automated TDM is shown in Figure 1.

## 3. TDM Implementation in Clinical Practice

The implementation of TDM should become a part of routine TB treatment monitoring, as recommended by WHO [67]. However, the usage and customs of performing TDM have not yet been standardized in clinical practice. In order to apply TDM in routine clinical practice, a proper understanding of its entire process, principles, and benefits is required. In this section, we discuss the following four steps in performing TDM:

1. Defining the case;
2. Obtaining the blood samples;
3. Measuring drug concentrations;
4. Interpreting the results.

### 3.1. Defining the Case

Before performing TDM, it is imperative to define the most beneficial case. Patients with the possibility of low or high exposure to drugs should receive TDM during their TB treatment [68,69]. The general indications for TDM are widely known as suspected dose-related toxicity, suspected noncompliance, acute drug overdose, chronic drug abuse, reduced kidney or liver function, potential interaction with other drugs, evaluation of absorption, diagnosing undertreatment, guiding withdrawal of therapy, and optimalization of treatment during early therapy or dosage changes [70]. Although the benefit of TDM remains arguable in TB treatment, several lines of evidence have indicated the importance of TDM for TB treatment, particularly the MDR-TB [19,67]. Furthermore, the new practical guidelines of the American Thoracic Society (ATS) include TDM for TB treatment [27]. The guideline specifically mentions the following clinical situations as those that may require determining plasma concentrations:

1. Poor response to tuberculosis treatment despite adherence and fully drug-susceptible *Mycobacterium tuberculosis* strain;
2. Severe gastrointestinal abnormalities: severe gastroparesis, short bowel syndrome, chronic diarrhea with malabsorption;
3. Drug–drug interactions;
4. Impaired renal clearance: renal insufficiency, peritoneal dialysis, critically ill patients on continuous renal replacement;
5. HIV infection;
6. Diabetes mellitus;
7. Treatment using second-line drugs.

Looking closely into the suggested clinical situations that require TDM, TDM should not be implemented only for patients with MDR-TB. DS-TB patients with uncommon clinical situations, relapse or even delay converters should be evaluated through TDM [71,72]. In short, patients with complex clinical conditions that may alter the PK characteristics of drugs, the elderly, and children, are subjects for TDM application [66,69]. To consider the contribution of age, comorbidities, ethnic group, and other covariates of IIV in PK and PD, the patient demographics, indications for TDM, dose regimens, and comedications should be collected accurately.

### 3.2. Obtaining the Blood Samples

After selecting the target patient for TDM, the second step is to decide which sample is to be collected for analysis. Current clinical practice is mainly based on plasma samples [69]. To obtain blood samples, it is important to note that time points are adequately selected to enable accurate optimization of the dose [20,21,73]. Different optimal sampling strategies to obtain adequate exposure according to specific anti-TB drugs have been developed in numerous studies [12,15,17,72]. However, 2-point schemes will typically be used (2 and 6 h after dose) [12,17]. The 2 h post-dose is used to obtain the peak concentration, while the 6 h post-dose is required to portray the possibility of delay absorption [15,17]. Importantly, both samples should be measured at a steady state.

### 3.3. Measuring Drug Concentrations

In measuring drug concentration, the selection of instrumentation is considered critical [14,20]. Several options are available, yet liquid chromatography coupled with mass spectrometry (LC-MS) is the ideal assay machine to measure drug concentration [14,20] due to its high sensitivity and specificity. Since the TB treatment regimen contains a combination of several drugs, first- or second-line anti-TB drugs can be measured in a single run, reducing the cost needed [74,75]. However, developing the method and running the LC-MS assay requires a highly skilled experimenter [76].

### 3.4. Interpreting the Results

After the drug concentrations are generated, the clinical pharmacologists/pharmacist will interpret the PK parameters and PD results. The dose will be adjusted by proportionally increasing or decreasing the dose as per the reference target range [18,45,73]. In anti-TB drugs, dose adjustment should be made based on both drug exposure and the minimum inhibitory concentration (MIC) of the *Mycobacterium* *tuberculosis* strain, in order to attain the optimal PK/PD target [12,14,20,77]. Nonetheless, in practice, MIC measurement is sometimes not performed. In this case, the therapeutic range using C_max_ was used as the target of evaluation [12,17]. Performing a follow-up of TDM at 1–2 weeks after dose adjustment is recommended to ensure that the PK/PD targets are reached, and that the patient’s condition is improved [69].

## 4. Evidence of Benefits

Ideally, TDM can provide a meaningful benefit if the results are available and interpreted within days [14,20]. Accordingly, the therapies can be adjusted efficiently. A previous prospective study reported the benefit of TDM [52]. In this trial, TB patients with comorbidities had low anti-TB drug levels and a longer time to culture conversion. Therefore, TDM was conducted by taking the samples 2 h after the dose. Low concentrations of RIF and INH were observed. A dose adjustment was made based on the physician’s discretion, after which the patients showed good improvement. This trial emphasized the importance of TDM in TB treatment. Not only does it improve the patient’s condition, but it also captures the evidence of underdosing of current anti-TB drugs [78,79]. A recent population PK study from our group also looked at INH dose and NAT2 acetylator status and highlighted that the current dose is not enough for rapid acetylators, but it is considerably high for slow ones [59]. These findings have strengthened the importance of individual dosing in order to reduce the development of drug resistance and avoid the risk of ADR. In this section, we summarized the studies that show how TDM was applied to the management of TB patients, the number of cases analyzed, countries usually performing TDM, characteristics of populations, and clinician responses to drug concentrations (Table 2). The presented studies clearly demonstrate the improvement of treatment outcome after dosage adjustment following TDM results for both normal and special populations. The previous studies took samples at 2 h after dose (C_2hr_), which in most anti-TB drugs can be assessed as C_max_. The concentrations were compared to the well-known therapeutic range of each drug, depending on how each study defined therapeutic range. INH and RIF were identified as the most frequent drugs with subtherapeutic concentrations. These results potentially contributed to patients having slow response to TB treatment, as both drugs are the backbone of therapy. Even though most of the studies adjusted the dose for each patient, a clear explanation of how the physicians corrected these doses is unavailable.

## 5. Barriers of Implementation in Clinical Practice

The application of TDM in the management of TB continues to face several barriers, such as the cost of analysis and shipping, laborious human resources, availability of TDM laboratories, and the availability of expert interpretation [9,16,47,67]. Therefore, we divided the current hurdles in TDM interpretation into four main categories.

### 5.1. Sampling Strategy 

To successfully perform TDM, it is critical to collect the appropriate sample at the appropriate time. The LSS used for targeting anti-TB drugs should be assessed based on available population PK data. The PK/PD target index for most anti-TB drugs is based on overall drug exposure (AUC) to the bacterial MIC [12,17]. Therefore, the application of TDM that only compares measured peak concentration and the therapeutic range should be discouraged. The integration of LSS with the population PK model or multiple linear regression can estimate reliable exposure for TDM practice [10,20,87]. This widely used LSS still has some obstacles in actual clinical settings. Patients often show up late to the clinic. Thus, the peak concentration time is often missed, and staying in the clinic for a long time in order to obtain serial sampling for TDM is inconvenient for patients. The sampling strategy for TDM should be flexible during actual clinical situations in order to be implemented successfully [58,88]. Another hurdle in terms of sampling strategy is the steady-state sampling required in conventional TDM [20]. Due to previous requirements, TDM is conducted after several days of treatment to enable the drug to obtain steady-state conditions, such as CFZ, EMB, PZA, and BDQ [12,15,68]. This delays the whole process significantly. In the case of infectious diseases, especially TB, the PK/PD target should be attained as early as possible [20]. In addition, plasma has been the most studied matrix over the past few decades in terms of TDM practice [17,50]. Nonetheless, it has disadvantages regarding the invasive techniques required, the instability of some drugs, and differences in drug concentrations in the target organ [20,72].

In our center (Center for Personalized Medicine of Tuberculosis (cPMTb)), we routinely perform TDM for TB patients. We employ MIPD-based TDM. A single point can be taken from patients while still allowing for precise exposure prediction before and after dose adjustments. We have successfully established the population PK model of INH, including NAT2 as the covariate, based on data taken using flexible sampling times [59,63]. The model can adequately predict the INH PK in each specific population. Moreover, the model can provide the recommended initial dose stratified by the NAT2 acetylator status [59]. These models show the proof-of-concept that the population PK model established using a convenient sampling strategy still shows reliable results in PK parameter prediction.

### 5.2. Logistic and Storage

Plasma and serum are the most widely used samples when measuring drug concentrations [69]. Most reference values for TDM have been established according to these matrices. The plasma or serum samples should be immediately harvested, frozen, and stored at −80 °C for subsequent analyses [69]. INH and ETA concentrations are widely affected by temperature [72]; they are not stable at room temperature and are prone to degradation [72]. As the highest-burden countries regarding TB are primarily located in resource-limited settings, this situation will be the main hurdle for logistical and transportation issues. Therefore, many methods have been developed to measure TDM samples in other body matrices, such as dried blood spots (DBS), interstitial space fluid (ISF), and saliva [89,90,91]. Of note, DBS has become the most recognizable alternative in the application of TDM [89]. DBS samples do not need to be frozen, as drug stability is usually much higher in the DBS than in a plasma sample [89].

### 5.3. Bioanalysis Process

Determining the concentration of drugs in biological samples can be accomplished via several different bioanalytical methods such as immunoassays, LC with fluorescence or ultraviolet detection, and LC-MS [14,20]. The application of LC-MS for quantifying drug concentrations has offered opportunities in sufficiently conducting TDM [14,20]. Nevertheless, the set-up and running of a dedicated TDM laboratory and the development of the analytical methods are expensive, time-consuming, complex, and require a high level of expertise [25,76]. Furthermore, most TB high-burden countries have limited access to costly equipment [67]. In our center, the bioanalysis of more than 22 anti-TB drugs can be measured simultaneously [74], reducing the cost and time needed to attain all drug concentrations for one TB patient.

### 5.4. Human Resources

The most crucial aspect of any TDM service is the expert clinical interpretation of drug concentration measurements from individual patients, whose doses will be adjusted accordingly [50,70,88]. To interpret TDM results, a clinical pharmacologist/pharmacist or clinician with further training in pharmacology is a prerequisite [73,88]. TDM encompasses consideration of plasma’s drug level, as well as the patient’s clinical outcome, drug characteristics, and medical history, to determine if a dose adjustment is necessary [70,88]. In addition, a clinical pharmacologist/pharmacist or clinician has to treat patients, not the plasma/serum levels [92]. However, highly skilled experts in this field remain scarce. In conventional TDM, the clinical pharmacologist/pharmacist needs to comprehensively evaluate the results of analysis for each patient following the therapeutic target of the specific drug. This process, of course, is enormously laborious and time-consuming. Additionally, many human resources are needed to conduct the TDM process, such as nurses to take the samples from the patient, laboratory workers to process the blood samples, and experts in bioanalysis [49].

## 6. Semi-Automated TDM Process

**Figure 2:**
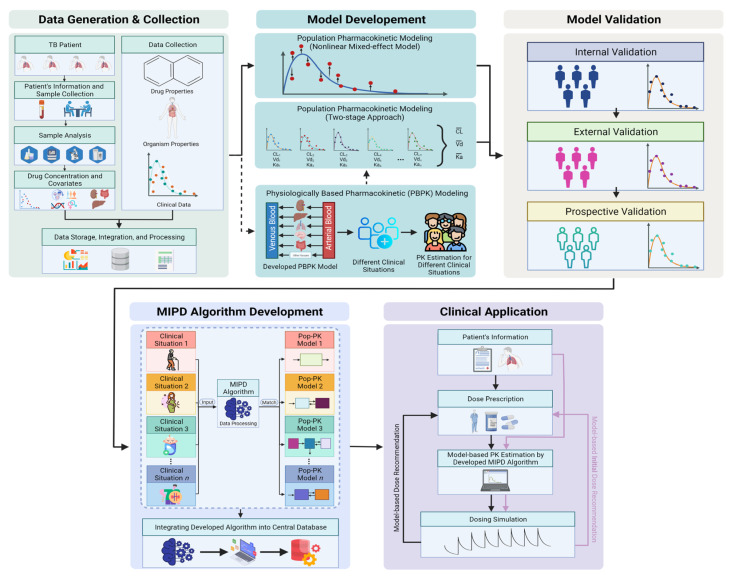
Overview of the semi-automated TDM workflow. TDM for TB treatment from bench to bedside application using integrated MIPD and clinical information within one central platform. The bold line for population PK modelling represents the current implementation of population PK based TDM in our workstation. The dashed line for PBPK shows the future perspective of PBPK implementation in a MIPD-based TDM approach. MIPD algorithm development shows that the developed model can be used in specific clinical situations. The pink line in clinical application represents the flow to provide the initial dose in our workstation. The black line in clinical application represents the semi-automated TDM process on our website. TDM: therapeutic drug monitoring; PK: pharmacokinetics; PBPK: physiologically based pharmacokinetics modelling; MIPD: model-informed precision dosing.

The semi-automated TDM process promises to implement personalized interventions by providing rapid, straightforward, and affordable clinical evaluations, which can immediately be translated into clinical decisions near the patient. This approach can be considered a bridge between MIPD and the clinical setting and can thus improve pharmacotherapy. This approach is called semi-automated TDM because earlier steps still need to be performed by human resources (defining the case, taking blood/plasma, and the genetic/bioanalysis process). However, the interpretation of analysis results, estimation of the individual PK, and dose recommendation are generated through the developed and validated system. Importantly, the PK interpretation allows for the evaluation of the overall exposure of drugs, not just C_max_ evaluation. Advanced technology could promote more accurate results and facilitate the development of a more holistic approach to anti-TB treatment. Thereafter, a dose recommendation is provided following the individual PK parameters and the therapeutic target reference. Based on the MIPD algorithm integrated into the central workstation, the patient’s data (demographic characteristics, genetic results, drug concentration results, and dose) are added; then, the previously established population PK model (incorporated with the significant covariate) is used for Bayesian forecasting. The Bayesian forecasting generates the PK profile for each individual and the models used are integrated into the user-friendly interface. Subsequently, a TDM report for each patient can be obtained from direct access to the workstation by the physician. The users (e.g., physicians) can select a particular drug to simulate the provided drug concentration. After the simulation process, a table with individual PK profiles and a figure containing the result of all simulated drug exposures is displayed. A basic interpretation of the result is also generated. Physicians can adjust the dose and dosing interval accordingly, corresponding with the results from the initial simulation (Figure 2). Users can navigate between the different results for comparison. All the results can be compiled into a report that the physicians can easily access at any time.

The goal of MIPD is to improve drug treatment outcomes in patients by achieving the optimal balance between efficacy and toxicity for the individual patient [4]. The approach is based on the available information about the patient and the disease that they are being treated for, comorbid diseases afflicting the patient, and the medications they are receiving [14]. The power of MIPD, especially in population PK modelling, depends on the number of patients included during model establishment [64]. It is also worth mentioning that the model developed based on a specific population provides a more precise result when implemented in the representative population [10]. The values of our proposed program and the models incorporated into the user interface were built on a large dataset and are available for first- and second-line anti-TB drugs. Indeed, it is not easy to collect thousands of datapoints in order to develop a more accurate model to make a prediction with a small margin of error. Subsequently, the covariate selected is more meaningful, since covariate identification is based on a large sample size, and the models evaluate both genetic and non-genetic covariates comprehensively. Therefore, the result adequately represents the whole population, especially the Korean one. To the authors’ knowledge, models developed using a large sample size remain scarce. Furthermore, our platform is callable and will eventually be cost-effective, considering its wide-reaching target populations. In addition, this is potentially helpful regarding introducing such an advanced technology to clinicians in high-TB-burden countries. Most available models for Bayesian forecasting were established based on Western countries, which usually have different characteristics to other populations [10,57]. By using this semi-automated TDM approach, the geographical barriers can be removed. In addition, our model has successfully demonstrated clinically relevant dose recommendation through the use of a time-flexible sampling strategy [59,63]. The strategy proposed may be one way to obtain cost-effective TDM by removing the necessity of expert validation and interpretation of the results. The scarcity of skillful clinical pharmacologists is an obstacle and one reason why TDM seems unpracticable in resource-limited countries [88]. In addition, this can encourage the cost effective drug prescription. By providing the initial dose before drug administration in complex situations, it can provide safe therapy. Moreover, therapy monitoring through MIPD does not need to wait until the drug reaches the steady state and can allow the adjustment of the dose at the earliest possible instance. Inadequate doses of anti-TB drugs create a financial burden for both individuals and governments due to the long period of treatment needed and expensive treatment of drug-resistant TB [14,19]. Therefore, personalized medicine facilitated through TDM is aimed at reducing the costs related to clinical management in the near future. However, it does not reduce the cost of the technical implications. Nonetheless, it still provides high-burden countries, such as Indonesia, with the ability to adapt to the concept of personalized medicine through the nation’s collaboration in developing a model based on their own ethnicity. The estimated PK, the identified significant covariate, and the suggested dose will be more accurate and clinically beneficial. Furthermore, our center also developed a method to measure the drug concentration from a DBS sample. The involvement of DBS in TDM has been considered a game-changer [69] and will provide easier methods for sample shipment, yield reasonable concentrations, and reduce the cost of sampling.

## 7. Future Perspectives

Precision medicine through TDM has shown that clinical characterization and treatment monitoring at the patient-level has the potential to reform TB treatment. For almost a decade, TB has remained the leading cause of mortality in infectious diseases [1]. COVID-19 has occupied recent global concern and funding due to its rapid spread [2] and has reversed the progress made in controlling TB globally [93]. All resources (hospital care, physicians, rapid testing devices) have been concentrated on fighting COVID-19. Therefore, this global pandemic has led to a substantial delay in TB treatment access for both outpatients and inpatients and has affected their care, including treatment monitoring and evaluation, which are essential in chronic disease [2,93].

MIPD has evolved as an important tool to streamline the TDM process and ensure effective treatment [20]. Typically, population PK is the most well-known tool in the practice of MIPD [14,68]. Population PK/PD models are based on direct measurement of drug exposure or efficacy obtained from the patient, recognized as real-time MIPD (data-driven approach) [4]. Many population PK models have been established for various anti-TB drugs [10,23,61,94]. Apparently, many of these models are assembled on limited datasets and their applicability is confined to patients with similar characteristics as the base model, such as ethnicities or disease states. Moreover, a model created directly from the subset edge population usually has a dataset with a small sample size. Population PK/PD models established using vulnerable populations such as pregnant women and children remain scarce [10]. In other words, population PK reaches its limits when specific patient populations are under-represented in the dataset and the simulations created for these populations will become biased. Another possible approach known as mechanistic MIPD has been proposed [4]. In recent years, the capacity of physiologically based pharmacokinetics (PBPK) modeling to evaluate physiological covariates associated with variability in drug exposure has gained attention [95,96]. PBPK models can be used for various purposes and applications and present numerous advantages compared to other methods [96,97]. PBPK models incorporate physiological data from preclinical species, aid in allometric scaling, can be parametrized for specific individuals or populations, and account for sequential metabolism- and permeability-constrained processes [95,97,98]. PBPK is a promising tool that may help apply more precise MIPD to edge populations. A previous study in PBPK of INH has suggested the need for acetylator-specific dose adjustments for optimal treatment outcomes [99]. Patients with slow acetylators should have their dose reduced to avoid the risk of ADR. This study showed that PBPK has the potential to eventually be adapted in the clinical use of TDM. In advance, PBPK and population PK have been used as a combined approach in several complimentary confirmation studies [95,100,101]. As far as we are concerned, PBPK can also be combined with population PK to implement Bayesian forecasting (middle-out approach). The capability of PBPK to generate data on virtual edge populations following specific clinical scenarios can be integrated into the two-stage approach/population PK model process to generate the typical PK value of those populations. This result is used to develop the MIPD algorithm for edge populations and is further included in Bayesian forecasting (Figure 2). Internal and external validation is used to validate the developed models. Likewise, in our cohort, the developed model is used to generate the PK parameters of anti-TB drugs and is further validated using prospective incoming TB patients by measuring the model’s predictive error. Thereafter, the full MIPD algorithm provides both initial doses, depending on the clinical situation, and dosage recommendation, following the result of semi-automated TDM. In cases where a physician is uncertain and may prescribe the initial dose (complex clinical conditions), they may select a model developed based on a similar clinical situation, fill in the patient’s characteristics, and obtain the result of individual estimation PK along with the initial dose.

Although the semi-automated TDM concept that we utilized is not first-time [55], this review underlines the importance of shifting the TDM approach from a complex and time-consuming strategy toward an advanced yet applicable integrated system, in order to support clinical decision-making. This novel strategy can be achieved by maximizing the capacity of available technological solutions, thus extending TDM benefits to anti-TB drugs that are not currently covered by routine monitoring. MIPD integrated into the clinical decision support system (CDSS) should be an indispensable tool to help clinicians adjust doses more easily. Our workstation for the personalized medicine of TB, named the cPMTb Smart R&D Workstation, stores patient information separately from the web interface for security reasons. Data come from different sources: patient demographics from clinicians/nurses, dose adjustments, PK/PD interpretation from clinical pharmacologists/pharmacists, and drug concentrations and genetic results from laboratory scientists. The cPMTb Smart R&D Workstation allows for the comprehensive evaluation of the patient’s condition within clinical teams. Therefore, practitioners can access the database and input and manage data according to their specific roles. Semi-automated TDM will gradually become fully automatic with more advanced technologies. It is possible that, in the future, TDM will be performed on portable devices, and the data will be transferred into a central database without human intervention (integration with electronic medical records) [25,49,102]. The COVID-19 pandemic has shown us the importance and benefits of telehealth [103]. During the last few years, telehealth has become a prominent tool regarding access to healthcare and this is anticipated to continue. Telehealth opens up opportunities to improve patient care and monitoring while minimizing the burden of logistics, cost, time consumed, and more importantly, removing geographical barriers [103]. Previously identified as strategies to end COVID-19, adequate infrastructure, healthcare systems, and funds to rapidly contain the outbreak were critical but primarily infeasible in developing countries [93]. This strategy should not be implemented only in the context of COVID-19, but also in TB management. Furthermore, we also would like to emphasize that the contribution of a single nation or center cannot aid in reaching the goal of “a world without TB” set by the WHO. The collaboration of multiple nations or centers, technical transfer, and data standardization and sharing are required to facilitate the eradication of TB. The proposed novel strategy has provided new hope for equality in accessing advanced technology to end the global issue of TB.

## Figures and Tables

**Table 1:** Evidence of altered drug exposure and its clinical relevance.

Clinical Relevance	Anti-TB Drug	Evidence
Inadequate drug levels may lead to a delay in culture conversion and treatment failure	RIF	Current standard dose of RIF has shown inadequate levels of RIF and may contribute to the treatment failure and relapse, high dose RIF has been evaluated and showed promising results for shortening the treatment duration and obtaining early bacterial conversion [28,33].
	PZA	Low concentration of PZA with a standard dose was associated with the delayed culture conversion, even though the DOTs had been implemented [34].
Low drug levels may acquire drug resistance	INH	NAT2 rapid acetylator has a faster clearance rate of INH from the liver, therefore reducing the plasma concentration and exposure of INH and eventually decreased sputum conversion rates and poorer microbiological outcomes [35,36]. Patients with rapid acetylator can mostly be found in patients with drug-resistant TB [37].
	RIF	Low exposure of RIF during the initial phase of therapy may put INH under monotherapy, which will eventually emerge as drug resistance [32].
High drug levels may cause adverse events	LZD	A previous study from China found that C_min_ of LZD was significantly higher in the patients with thrombocytopenia (C_min_ = 8.81 mg/L, *p* < 0.0001) [38].
		Another study from Taiwan reported that the C_min_ and AUC_0–24 h_ of LZD in patients with thrombocytopenia were significantly higher (C_min_ = 13 mg/L and AUC_0–24 h_ = 451 mg·h/L) [39].
	PZA	Pyrazinoic acid, as an active metabolite of PZA, increases serum uric acid based on its trans-stimulatory effect on URAT1, causing the reabsorption of urate from the luminal side into tubular cells and eventually hyperuricemia [40,41].
		The accumulated metabolite concentrations of pyrazinoic acid and 5-hydroxy-pyrazinoic acid have been linked to the PZA-induced liver injury [42].
	INH	Although it remains arguable, high concentrations of INH also may increase the risk of drug-induced liver injury in slow acetylator patients due to slow clearance rate of INH from liver [43,44].

RIF: Rifampicin; PZA: Pyrazinamide; INH: Isoniazid; LZD: Linezolid; NAT2: N-acetyltransferase 2; DOTs: Direct observed therapy, short-course; URAT1: human urate transporter 1; C_min_: minimum concentration; AUC_0–24h_: area under curve from 0 to 24 h; TB: tuberculosis.

**Table 2:** Studies showing benefits of TDM in TB management.

Author	Country	Study Design	Population Characteristics	Cases (n)	Drugs Measured	TDM Results	Conclusion
Heysell et al. (2010) [51]	USA	Retrospective cohort	DS-TB, 42 slow response patients 269 normal patients	311	RIF: 600mg INH: 300 mg PZA and EMB: weight based daily dose.	Median C_2hr_ [IQR], μg/mL INH: 1.9 (1.1–3.5) RIF: 7.4 (2.5–11.4) PZA: 2.5 (1.7–3.2) EMB: 28.1 (26.5–33.2) Proportion of patients with low C_2hr_, (lower limit of therapeutic range, μg/mL)_:_ INH: 33% (<3) RIF: 33% (<8) PZA: 0% (<20) EMB: 31% (<2)	Subtherapeutic concentrations of RIF, INH, and EMB were frequently observed, dosage adjustment for INH and RIF from 300 mg and 600 mg daily to 450 mg and 900 mg daily. For intermittent INH interval, the dose was increased from 900 mg to 1200 mg. DM was associated with slow response and low RIF concentrations. Patients with TDM have 2 months shorter therapy.
Babalik et al. (2011) [52]	Canada	Retrospective case-control	DS-TB, 20 cases (TDM done) 20 controls (no TDM) 8 with HIV (all cases)	40	INH: 5 mg/kg, max 300 mg RIF: 10 mg/kg, max 600 mg PZA: 20 mg/kg EMB: 15 mg/kg RFB: 0.8 ± 0.3 mg/kg	Mean C_2hr_ ± SD, (μg/mL) INH: 2.0 ± 1.3 RIF: 9.1 ± 4 RFB: 0.2 ± 0.1 PZA: 32.9 ± 11.3 Proportion of patients with low C_2hr_, (lower limit of therapeutic range, μg/mL)_:_ INH: 87% (<3) RIF: 67% (<8) RFB: 89% (<0.3) PZA: 15% (<20)	Subtherapeutic concentrations of RIF, RFB, and INH were frequently observed. Mean dosage adjustment ± SD, (mg/kg): INH: 8.1 ± 1.8 RIF: 13.5 ± 1.7 RFB: 2.5 ± 0.9 PZA: 25.5 ± 12.6 Low concentration was found mostly in HIV patients. Subtherapeutic concentrations associated with longer therapy duration.
Kayhan et al. (2011) [80]	Turkey	Prospective observational cohort	DS-TB, patients excluded: HIV and DM	49	INH: 300 mg RIF: 600 mg PZA: 1500 mg or 2000 mg (weight adjusted) EMB: 1000 or 1500 mg (weight adjusted)	Mean C_2hr_ ± SD, (μg/mL) INH: 3.83 ± 2.09 RIF: 6.13 ± 4.27 PZA: 32.2 ± 16.96 EMB: 3.68 ± 2.41 Proportion of patients with low C_2hr_, (lower limit of therapeutic range, μg/mL)_:_ INH: 29% (<3) RIF: 74% (<8) PZA: 20% (<20) EMB: 18% (<2)	Subtherapeutic concentrations of RIF and INH were frequently observed, dosage adjustment was performed in low serum drug concentrations.
Magis-Escurra et al. (2012) [18]	Netherlands	Retrospective case series	Relapse TB, delayed converter	4	RIF, INH, PZA, EMB (doses were not described clearly)	Patient 1 C_2h_, (lower limit of therapeutic range, μg/mL): RIF: 5.6 (<8) INH: <0.025 (<3) PZA: 8.3 (<20) Patient 2 C_2h_, (lower limit of therapeutic range, μg/mL): RIF: 4.1 (<8) Patient 3 C_2h_, (lower limit of therapeutic range, μg/mL): RIF: 4.0 (<8) PZA: 10.0 (<20) Patient 4 C_2h_, (lower limit of therapeutic range, μg/mL): RIF: 2.3 (<8)	Subtherapeutic concentration of RIF associated with delayed conversion, dosage adjustment of: Patient 1: RIF from 600 mg to 1200 mg INH and PZA also were adjusted. Patient 2: RIF from 600 mg to 1200 mg Patient 3: INH from 200 to 250 mg, RIF from 450 to 600 mg, PZA from 1250 to 2000 mg Patient 4: RIF from 600 to 900 mg (not enough to reach the target concentration) then increased to 1200 mg The dose adjustment for all patients improved the treatment outcomes and increased the C_2h_ of RIF to achieve therapeutic target.
Heysell et al. (2013) [81]	USA	Retrospective cohort	TB-DM: 21 patients TB-slow responders: 14 patients	35	RIF: 600 mg INH: 300 mg INH (intermittent): 900 mg	Mean C_2hr_ ± SD, (μg/mL): *Daily* INH_DM_: 2.0 ± 1.3 INH_slow:_ 3.1 ± 1.1 RIF_DM_: 6.6 ± 4.3 RIF_slow_: 8.2 ± 6.2 *Intermittent* INH_DM_: 6.0 ± 3.0 INH_slow:_ 11.3 ± 2.5 Proportion of patients with low C_2hr_, (lower limit of therapeutic range, μg/mL)_:_ Daily INH_DM_: 65% (<3) INH_slow_: 63% (<3) RIF_DM_: 60% (<8) RIF_slow_: 41% (<8) *Intermittent* INH_DM_: 75% (<9) INH_slow_: 17% (<9)	Subtherapeutic concentrations of RIF and INH were frequently observed, dosage adjustment for INH and RIF from 300 mg and 600 mg daily to 450 mg and 900 mg daily. For intermittent INH dose from 900 mg was increased to 1200 mg. DM is associated with subtherapeutic concentration of RIF and INH. Early dose correction using TDM decreased the number of slow responders.
Mehta et al. (2001) [82]	USA	Retrospective case series	DS-TB, slow response to treatment HIV: 1 patient	6	RIF: 600 mg INH: 300 mg PZA: 25 mg/kg EMB: 25 mg/kg	Patient 1 C_2h_, (lower limit of therapeutic range, μg/mL): RIF: 1.5 (<8) Patient 2 C_2h_, (lower limit of therapeutic range, μg/mL): RIF: 5.9 (<8) Patient 3 C_2h_, (lower limit of therapeutic range, μg/mL): RIF: <1.0 (<8) Patient 4 C_2h_, (lower limit of therapeutic range, μg/mL): RIF: <1.0 (<8) Patient 5 C_2h_, (lower limit of therapeutic range, μg/mL): RIF: <1.0 (<8) Patient 6 C_2h_, (lower limit of therapeutic range, μg/mL): RIF: 3.54 (<8)	Subtherapeutic concentrations of RIF were observed in all patients, dosage adjustment was performed from 600 mg to 900 mg, one patient adjusted to 1500 mg (Patient 4). Dose adjustment improved the response of the patients.
Ray et al. (2003) [83]	Australia	Prospective cohort	DS-TB	90	RIF: 150, 300, 450, 600 and 750 mg, daily or 3 times weekly INH: 150, 200, 300, 350, 400, 450, 500, 600, and 750 mg daily or 3 divided-dose, weekly	Mean C_2hr_ ± SD, (μmol/L) INH: 11.1 ± 7 RIF: 28.5 ± 20.4 Proportion of patients with low C_2hr_, (lower limit of therapeutic range, μmol/L)_:_ INH: 46% (<22) RIF: 48% (<10) Proportion of patients with high C_2hr_, (upper limit of therapeutic range, μmol/L)_:_ INH: 29% (>37) RIF: 2% (>29)	High concentration of INH related to ADR and low concentration related to therapeutic failure. A case report of a slow converter that needed dosage adjustments of INH and RIF from 300 mg and 450 mg to 400 mg and 600 mg was presented. Sputum smear was improved after dose adjustment.
Heysell et al. (2015) [84]	USA	Retrospective cohort	MDR-TB, DM: 1 patient HIV: 1 patient	10	CAP: 15 mg/kg dose (maximum 1 g) MFX: 400 mg daily CS: 250 mg daily LZD: 400–600 mg AMK, PAS, EMB, PZA, ETA (doses were not described clearly for these drugs)	Mean C_2hr_ ± SD, (μg/mL): Daily CAP: 21.5 ± 14.0 AMK: 35.3 ± 3.7 MFX: 3.2 ± 1.5 CS: 16.6 ± 10.2 PAS: 65.0 ± 29.1 (C_6hr_) LZD: 11.4 ± 4.1 EMB: 1.8 ± 1.85 PZA: 39.9 ± 1.8 ETA: 1.5 Proportion of patients with low C_2hr,_ (lower limit of therapeutic range, μg/mL): CAP: 60% (<35) AMK: 50% (< 35) MFX: 20% (<3) CS: 57% (<20) PAS: 0 LZD: 33% (<12) EMB: 33% (<2) PZA: 0% (<20) ETA: 0% (<1)	Subtherapeutic concentrations were frequently observed in CAP, AMK, and CS. The doses were adjusted in CAP, MFX, CS, LZD, EMB (increased), and PZA (decreased). The outcome resulted in patients being cured or clinically improved.
Prahl et al. (2014) [85]	Denmark	Prospective observational study	DS-TB, HIV: 2 patients	32	INH: 5 mg/kg, max 300 mg RIF: 10 mg/kg, max 600 mg PZA: 30 mg/kg, max 2000 mg EMB: 20 mg/kg, max 1200 mg	Median C_2hr_ (range), μg/mL INH: 2.1 (0.5–12.1) RIF: 6.5 (0–31) PZA: 31.3 (14.9–110.2) EMB: 2.2 (0.5–5.9) Proportion of patients with low C_2hr_, (lower limit of therapeutic range, μg/mL): INH: 71% (<3) RIF: 58% (<8) PZA: 10% (<20) EMB: 46% (<2)	Subtherapeutic concentrations of RIF and INH were frequently observed, dosage adjustment for the low concentration drugs. Low INH and RIF C_2hr_ associated with poor outcome.
Hammi et al. (2016) [86]	Morocco	Retrospective case series	DS-TB, Delayed converter	4	Patient 1: RIF: 600 mg INH: 300 mg PZA: 1600 mg EMB: 1100 mg Patient 2: RIF: 450 mg INH: 225 mg PZA: 1200 mg EMB: 825 mg Patient 3: RIF: 450 mg INH: 225 mg PZA: 1200 mg EMB: 825 mg Patient 4: Unknown doses of RIF/INH/PZA/EMB	Patient 1 C_2h_, (lower limit of therapeutic range, μg/mL): RIF: 2.9 (<8) Patient 2 C_2h_, (lower limit of therapeutic range, μg/mL): RIF: 4.8 (<8) Patient 3 C_2h_, (lower limit of therapeutic range, μg/mL): RIF: 3.85 (<8) INH: 0.59 (<3) Patient 4 C_2h_, (lower limit of therapeutic range, μg/mL): RIF: 1.79 (<8)	Subtherapeutic concentration of RIF associated with delayed conversion, dosage adjustment of: Patient 1: RIF from 600 mg to 855 mg. Patient 2: RIF from 450 mg to 750 mg. Patient 3: INH from 225 to 300 mg, RIF from 450 to 600 mg, Patient 4: RIF dose was increased. The dose adjustment for all patients improved the treatment outcomes and increased C_2h_ of RIF to achieve therapeutic target.

DS-TB: drug susceptible tuberculosis; MDR-TB: multi-drug resistant tuberculosis; RIF: Rifampicin; INH: Isoniazid; EMB: Ethambutol; PZA: Pyrazinamide; RFB: Rifabutin; CS: Cycloserine; ETA: Ethionamide; CAP: Capreomycin; AMK: Amikacin; LZD: Linezolid; MFX: Moxifloxacin; PAS: Para-aminosalicylic acid; HIV: human immunodeficiency virus; C_2h_: Concentration at 2 h after dose; C_6hr_: Concentration at 6 h after dose; DM: Diabetes Mellitus; IQR: inter quartile range; SD: standard deviation; TDM: therapeutic drug monitoring.

## Data Availability

Not applicable.
